# Greenhouse Gas Emissions from Calf- and Yearling-Fed Beef Production Systems, With and Without the Use of Growth Promotants

**DOI:** 10.3390/ani2020195

**Published:** 2012-04-16

**Authors:** John Basarab, Vern Baron, Óscar López-Campos, Jennifer Aalhus, Karen Haugen-Kozyra, Erasmus Okine

**Affiliations:** 1 Alberta Agriculture and Rural Development, Lacombe Research Centre, 6000 C & E Trail, Lacombe, AB T4L 1W1, Canada; 2 Agriculture and Agri-Food Canada, Lacombe Research Centre, 6000 C & E Trail, Lacombe, AB T4L 1W1, Canada; E-Mails: vern.baron@agr.gc.ca (V.B.); Oscar.LopezCampos@ agr.gc.ca (O.L.-C.); Jennifer.Aalhus@agr.gc.ca (J.A.); 3 The Prasino Group, 12207-42 A Avenue, Edmonton, AB T6J 0X5, Canada; E-Mail: karenhk@prasinogroup.com; 4 Department Agricultural, Food and Nutritional Science, University of Alberta, Edmonton, AB T6G 2P5, Canada; E-Mail: Erasmus.Okine@ales.ualberta.ca

**Keywords:** beef, life cycle assessment, carbon footprint, hormone implant, production efficiency

## Abstract

**Simple Summary:**

A spring calving herd (~350 beef cows) over two production cycles was used to compare the whole-farm greenhouse gas (GHG) emissions among calf-fed *vs.* yearling-fed production systems, with and without growth implants. Farm GHG emissions initially included enteric CH_4_, manure CH_4_ and N_2_O, cropping N_2_O, and energy use CO_2_. The carbon footprint ranged from 19.9–22.5 kg CO_2_e per kg carcass weight. Including soil organic carbon loss from annual cropping and carbon sequestration from perennial pastures and haylands further reduced the carbon footprint by 11–16%. The carbon footprint of beef was reduced by growth promotants (4.9–5.1%) and by calf-fed beef production (6.3–7.5%).

**Abstract:**

A spring calving herd consisting of about 350 beef cows, 14–16 breeding bulls, 60 replacement heifers and 112 steers were used to compare the whole-farm GHG emissions among calf-fed *vs.* yearling-fed production systems with and without growth implants. Carbon footprint ranged from 11.63 to 13.22 kg CO_2_e per kg live weight (19.87–22.52 kg CO_2_e per kg carcass weight). Enteric CH_4_ was the largest source of GHG emissions (53–54%), followed by manure N_2_O (20–22%), cropping N_2_O (11%), energy use CO_2_ (9–9.5%), and manure CH_4_ (4–6%). Beef cow accounted for 77% and 58% of the GHG emissions in the calf-fed and yearling-fed. Feeders accounted for the second highest GHG emissions (15% calf-fed; 35–36% yearling-fed). Implants reduced the carbon footprint by 4.9–5.1% compared with hormone-free. Calf-fed reduced the carbon footprint by 6.3–7.5% compared with yearling-fed. When expressed as kg CO_2_e per kg carcass weight per year the carbon footprint of calf-fed production was 73.9–76.1% lower than yearling-fed production, and calf-fed implanted was 85% lower than hormone-free yearling-fed. Reducing GHG emissions from beef production may be accomplished by improving the feed efficiency of the cow herd, decreasing the days on low quality feeds, and reducing the age at harvest of youthful cattle.

## 1. Introduction

The efficient use of energy, land and water will continue to be a challenge for agriculture as the global population increases to a predicted 9.5 billion people by 2050, food requirements increase by 70% compared with present day and resources available for agricultural production decrease [1]. In the past the livestock industries have met these challenges through improved productivity, resulting in more milk or meat in a set period of time per unit of animal input [2,3,4]. Improved productivity allows the livestock industry to reduce resource use and waste outputs, primarily in the form of manure and greenhouse gas (GHG) emissions (CO_2_, CH_4_ and N_2_O), through the “dilution of maintenance” effect since every animal has a maintenance nutrient requirement that must be fulfilled before nutrients are available for production [3]. In the beef cow, energy for maintenance represents 65–75% of total feed energy requirements [5] and 56–71% of the cost of cow-calf production is associated with feed, bedding and pasture [6]. Improved productivity has occurred due to advances in nutrition and ration formulation, herd fertility, vaccines and animal health, genetic selection, pasture management, growth promotants and feed additives (e.g., *β*-adrenergic agonist), resulting in a 16% decrease in the carbon footprint per unit of beef [2,3]. However these conventional, more intensive beef production systems continue to be challenged by media and public perception as having negative impacts on the environment, and that more extensive production systems associated with labels such as natural, organic, hormone-free, and grass-finished would have a lower carbon footprint and be more sustainable. Contrary to this belief, conventional beef production systems are consistently reported to have a lower carbon footprint and use less feed, water and land than natural or grass-finished beef productions systems [2,3,4,7]. Globally the carbon footprint of beef (kg CO_2_e kg^−1^ of carcass weight) varies widely from as low as 10 for feedlot finished beef in Australia [8] to as high as 44 for grass-finished beef in Brazil that included land use change from deforestation of the rainforest [9]. In Canada primary scope life cycle assessment of GHG emissions from beef production are estimated at 17–22 kg CO_2_e kg^−1^ carcass weight [10,11].

Despite these improvements in production considerable reductions are still possible due to inherent inefficiency within the North American beef production system and the finding that maintenance requirements and feed efficiency of beef cattle have remained largely unchanged over the last 100 years [12,13,14]. In contrast competing protein sources such as pork and poultry have made dramatic improvements in feed efficiency through both genetic and non-genetic means [15,16,17,18]. Hume *et al.* [4] recently reported that genetic improvements in layers, broilers, pigs and dairy have decreased methane and nitrous oxide emissions by 14–30%, while genetic improvement in beef and sheep have resulted in little to no reduction of methane and nitrous oxide emissions per unit of product. Further, an analysis of 1.7 million records from the Canadian Cattle Identification Agency (CCIA) data base revealed that the average age at slaughter in Canada was estimated at 18.6 months of age as of 1 June 2009, and 39.5% of the cattle were harvested between 19 and 25 months of age [19], suggesting considerable opportunity to reduce the age at slaughter in youthful beef cattle, which may improve system efficiency and lower the carbon footprint of beef.

In Canada few studies have examined the impact of different management strategies and beef production systems on GHG emissions and most predict dry matter intake (DMI) within cattle class and feeding period based on NRC [20] and IPCC [21,22] equations and cropping GHG emission coefficients are taken from the literature [10,11]. In addition, Alberta is the first province in Canada to legislate GHG emission reduction (1 July 2007; http://environment.alberta.ca/0915.html) and have all companies that emit more than 100,000 tonnes of GHG a year reduce their emission intensity by 12% per year compared to a baseline [19]. Companies have the option to purchase carbon offset credits that have followed a government-approved quantification protocol listed at http://environment.alberta.ca/02275.html.

One of the approved beef protocols listed is “Reduced days at harvest in beef cattle”. This protocol is based on IPCC [22] equations and theoretical calculations of reductions. Thus, the purpose of this study was to conduct a primary scope life cycle assessment of beef cattle production for GHG emissions using actual feed, energy and cropping inputs and beef outputs from calf-fed *vs.* yearling-fed production systems with, and without, aggressive growth implant.

## 2. Experimental Section

### 2.1. System Boundary and Scope

This study used ISO-compliant life cycle assessment to compare the cradle-to-farm gate cumulative GHG emissions associated with four beef production strategies. The spring calving herd consisting of approximately 350 cows and related feedlot operations at the Agriculture and Agri-Food Canada, Lacombe Research Centre (Lacombe, AB, Canada) were used for the collection of diet ingredient and nutrient composition, feed intake, and cropping inputs and outputs from the four beef production strategies. Beef production practices in this herd are typical for western Canada and are described in detail by Basarab *et al.* [23,24]. The herd consists of Hereford-Aberdeen Angus and Charolais-Red Angus crossbred cows which are also common breeds in western Canada [25]. The cattle used for the GHG assessment included cows, breeding bulls, replacement heifers, replacement bulls, calves from birth to weaning, and feeders from weaning to slaughter. All animals were maintained and cared for according to the guidelines of the Canadian Council on Animal Care [26].

Sources of GHG included on-farm emissions of CH_4_ from enteric fermentation and manure, on-farm emissions of N_2_O from manure, off-farm emissions of N_2_O from N leaching, run-off and volatilization, on-farm emissions of N_2_O from cropping due to soils, fertilizer, roots and residue, and CO_2_ emissions from energy use. Energy-use direct and indirect CO_2_ emissions included: (1) embodied energy in equipment for field operations, baling, hauling, feed processing, feeding, bedding and manure removal; (2) crop inputs such as fertilizer, herbicide and seed; and (3) fuel and lubrication for field operations, baling, hauling, feed processing, feeding, bedding and manure removal. All feeds except for the protein supplement were grown on-farm, thus CO_2_ emissions due to transporting feeds was negligible. All gases were expressed as CO_2_ equivalents (CO_2_e) to account for the global warming potential of each gas compared to CO_2_, where CO_2_ = 1, CH_4_ = 23 and N_2_O = 298 [21]. The carbon footprint or GHG intensity of beef was expressed as kg CO_2_e kg^−1^ live weight, CO_2_e kg^−1^ carcass weight, kg CO_2_e kg^−1^ live weight yr^−1^, CO_2_e kg^−1^ carcass weight yr^−1^.

### 2.2. Description of the Beef Production Systems

The life cycle assessment for cows, bulls, replacement heifers and feeder cattle followed the production cycle of the cow herd and began in late May of each year when the cow-calf pairs were placed on pasture prior to the beginning of the breeding season and ended 365 d later since the objective of cow-calf management is to calve once every year. Two production cycles were followed for all cattle categories. Numbers of animals followed in each cattle category, feeding period and production cycle varied and are presented in Table A1 and Table A2. Feed sampling, dry matter disappearance and quality analysis during the summer and fall grazing periods are described by López-Campos *et al.* [27]. For the remainder of the production cycle, diet ingredient composition and feed delivered to each cattle category were recorded daily by a feed truck (International DT4600 truck, Cattlelac 520 mixer, Weigh Tronic scale with onboard laptop) and later transferred to a daily time-step data base in Excel (Microsoft). Average diet ingredient composition, total digestible nutrients (TDN), crude protein (CP), body weight, ADG and DMI for cows, bulls and replacement heifers by feeding period are given in Table A1.

Feeder steers from the herd described above were used to create four beef production strategies: (1) hormone free cattle harvested at 11–14 mo of age, (2) growth implanted cattle harvested are 11–14 mo of age, (3) hormone free cattle harvested at 19–23 mo of age, and (4) growth implanted cattle harvested at 19–23 mo of age. These four production strategies were created because they represented the majority of youthful cattle, aged 10 to 24 mo of age, processed through Canadian packing plants [19]. Feeders harvested at 11–16 mo of age are referred to as “calf-fed” while those harvested at 17–23 mo of age are referred to as “yearling-fed”, which reflected the age at which the feeders are started on their finishing diet. In each of two years (2008 and 2009), 112 spring-born crossbred steer calves were equally assigned at weaning to two production systems (calf-fed; yearling-fed) and two implant groups (not implanted; implanted) based on breed cross, birth date, calf weight and dam age. In each year half the calf-fed steers (n = 28) were implanted with 200 mg progesterone and 20 mg estradiol benzoate (Component E-S, Elanco-Animal Health A Division of Eli Lilly Canada Inc., Toronto, ON, Canada) at weaning, and re-implanted with 120 mg trenbolone acetate and 24 mg estradiol (Component TE-S, Elanco-Animal Health A Division of Eli Lilly Canada Inc., Toronto, ON, Canada) approximately 90–100 d before slaughter. Similarly, half the yearling-fed steers each year (n = 28) were implanted at weaning and then four more times at 80–90 d intervals with 200 mg progesterone and 20 mg estradiol benzoate, and then with 120 mg trenbolone acetate and 24 mg estradiol 90–100 d before slaughter. A more detailed description of the animal management and experimental design can be obtained from López-Campos *et al.* [27]. The average diet ingredient and nutrient composition, body weight, ADG and DMI for the feeders by feeding period are given in Table A2. Since no feeder heifers were followed in the present study, it was assumed that the diet ingredient and nutrient composition for heifers was the same as for steers, and heifers had a 5% lower weaning weight, 10% slower post-weaning growth rate and 8% higher feed to gain ratio from weaning to slaughter compared with steers [23,28]. Both calf-fed and yearling-fed steers were targeted to be slaughtered at 8–10 mm of backfat in four groups of 14 per year. In 1–2 weeks intervals steers were trucked 3 km for processing at the Lacombe Research Centre abattoir. The values in Table A1 and Table A2 were then used to calculate the daily enteric CH_4_ emissions and emissions of CH_4_ and N_2_O from manure handling, storage and land application for each class of cattle and feeding period based on the IPCC [22] Tier 2 methodology and modified for nitrogen excretion according to NRC [20]. Table A1 and Table A2 values were also used to calculate the total amount of feed ingredients delivered to each cattle category (kg DM hd^−1^ d^−1^).

### 2.3. Description of Climate and Location

The research was located near Lacombe, AB, Canada (52°27'23''N, 113°44'31''W). The actual whole-farm location is the Agriculture and Agri-Food Canada Research Centre farm, which is representative of Ecodistrict 737 in the Parkland ecoregion [29]. The soil is Orthic Black Chernozem with clay and sandy loam textures. The topography is moderately undulating. The long term average precipitation is 450 mm annually and 341 mm within the May to October growing season. The ecodistrict precipitation to potential evapotranspiration ratio (P/PE) is 0.65 for the growing season based on 1970 to 2000 annual weather data normal. Growing season precipitation during 2008, 2009 and 2010 was 311, 243, and 513 mm, respectively. Temperatures both during the growing season and during winter were generally slightly cooler than average (Table A3).

### 2.4. Description of Crop and Pasture Complex

Crop production as required to feed all cattle classes was carried out on a farm scale. None of the land used could be classified as marginal. There was no summer-fallow. All pasture could be defined as cropland pasture in rotation with cereal and oilseed crops and not rangeland. Species varied between years, but field methods and crops used for feed were typical of the region. In 2008 barley (*Hordeum vulgare* L.) was used as silage and grain as feeds and straw for bedding; oat (*Avena sativa* L.) grain was used as feed. Summer pastures for cows consisted of perennial stands composed of largely meadow bromegrass (*Bromus riparius* Rehm.), but smooth bromegrass (*Bromus inermis* Leyss.) and Kentucky bluegrass (*Poa pratensis* L.) were also present depending on paddocks. Pastures had originally been sown as a mixture with alfalfa (*Medicago sativa* L.), but legume content was 20% or less. All of these pastures were over 10 yr-old. Pastures used for weaned calves and yearling stocker animals were planted in 2001 and consisted of meadow bromegrass and alfalfa with alfalfa less than 30% by 2008; Kentucky bluegrass existed in patches. In 2009 feed consisted of barley silage, grain and straw as well as straw for bedding. Pastures consisted of the same species mix, but areas used for grazing cows in summer were not identical.

Barley and oat were managed identically from year to year, although fertilizer inputs varied between the years. Tillage could be described as minimum or reduced as opposed to zero-tillage or conventional tillage. Seeding occurred at recommended rates. In 2008 fertilizer was broadcast prior to seeding at 100 kg N as urea, 30 kg P_2_O_5_ and 30 kg K_2_O ha^−1^ and in 2009 at 80 kg N and 20 kg P_2_O_5_ ha^−1^. All barley and oat crops received 1.236 L ha^−1^glyphosate [N-(phosphonomethyl) glycine] as a pre-seeding burn-off. In addition crops used for grain production were applied with a mixture of dicamba, [3,6-dicloro-(2-methoxybenzoic acid)] mecoprop {2-[4-chloro-(2-methylphenoxypropoanoic acid)]} and MCPA amine [4-chloro-(2-methylphenoxy) acetic acid] at 1.24 kg ha^−1^ total active ingredient. In addition roller-packing after seeding, swathing, combining and grain hauling activities were taken into account. Straw removal for bedding and feed were assumed to occur on all cereals harvested for grain. Thus, baling and hauling of large round bales occurred after combining. Because barley and oat grain and straw were used for different livestock classes within the four production strategies and in some cases in different enterprises (e.g., could be sold off the farm), emissions (kg CO_2 equiv_ ha^−1^) were divided between grain and straw in a 0.55 and 0.45 proportion according to crop dry matter distribution of the crop [30]. Barley silage activities included swathing, chopping, hauling and packing for a bunker silo.

Perennial hay, silage and pasture included inputs and operations for establishment. Establishment included seed at recommended rates for a 1:1 mixture of meadow bromegrass and alfalfa and no herbicide application or cover crop. Annual hay production included fertilizer broadcast, hay cutting with a haybine, baling and hauling. Hay and silage were included in the diets in 2008–09 only. Fertilizer inputs were 100 kg N as urea, 30 kg P_2_O_5_ and 30 kg K_2_O ha^−1^. Energy of all inputs and operations for hay and silage establishment were averaged over eight yrs, the expected life of the stand and 12.5% of energy or emission for establishment year was added to the total of annual hay and silage production. Pastures used for cows, calves and bulls during summer were assumed to have a 20-yr life span. Therefore establishment emissions of 5% of the establishment year total were added to those from annual input and operations. The cow-herd was grazed in two breeding groups of approximately equal size in different grazing cells. Cows were managed as in a controlled rotational grazing method, but rarely returned to any paddock for a second grazing. Carrying capacity averaged between the two herds within years was equivalent to 68 and 101 cow-calf pairs-d ha^−1^ with bull size and grazing days accounted for. No fertilizer was applied to these pastures in 2008. In 2009 either 42 kg N or 58 kg N ha^−1^ was applied to pastures for respective groups. Operations for fertilizer spreading, and moving and monitoring cows and calves while on pasture were recorded by time required for laborers and all terrain vehicle used.

Pasture used for weaned calves was similar to that used for yearling stocker animals and assumed to have an 8-yr life span. Therefore establishment emissions calculated as 12.5% of total emissions from the establishment year were added to annual emissions for both of these pasture-types. However, pastures used for weaned calves were used for a short period in each fall and had been used for hay production during the first cut. All calves weaned were co-mingled on the same pastures and managed identically. Therefore fertilizer added to these pastures was assumed to contribute only to the hay enterprise and not to the weaned calves. Carrying capacity for weaned calves was 560 and 550 animal-d ha^−1^ over 50 and 26 pasture days during 2008 and 2009, respectively. Operations related to monitoring, moving and bedding were included in emissions attributed to these animals. Pastures used for yearlings were managed as controlled rotational grazing using an electrified wire that was moved at least twice per week. Carrying capacity of steers was 115 and 160 animal-d ha^−1^ with 59 and 72 pasture days in 2009 and 2010, respectively. Fertilizer as 52 kg N ha^−1^ and 75 kg N ha^−1^ was broadcast in the spring of 2009 and 2010, respectively.

Average yields (kg DM ha^−1^) for all crops and feeds were determined as harvested yields of grain, straw or whole-plant forage on a field basis by recording truck-loads with representative fresh and dry weights. Where this was not possible, within field representative quadrat sampling (kg DM m^−2^) was extrapolated. All pastures were monitored using a cage method for available forage and residue yields on a dry matter basis as animals moved from paddock to paddock as was the case for cows and calves, or as electrified wire was moved within paddocks as was the case for weaned calves and yearlings. Duration of measurement period in the case of cow-pasture was no longer than 2 wks or less as cows moved from paddock to paddock more frequently. Concentrations of nitrogen (N) and carbon (C) were determined using a Leco C and N Analyser [31] on sub-sampled dried and ground subsamples. 

Sources of CO_2_ emission derived from on-farm energy use are summarized in Table A4. All energy used in manufacture and transportation of equipment (embodied), operation and maintenance (fuel and lubrication) was accounted for in all cropping activities for each crop species and cropping or pasture enterprise. Energy (MJ ha^−1^) was converted to diesel fuel equivalent (L ha^−1^) and then to CO_2_e (kg CO_2_e ha^−1^) [32]. Equipment used for each crop or animal management activity was referenced to Nagy [33] and Saskatchewan Agriculture Farm Machinery Custom Rental Guide 2008–09 [34] to determine a work rate (ha h^−1^) for the actual equipment combination used or its’ equivalent size and type. Then, for cropping activities, the equipment combination was matched to embodied fuel and lubrication energy required hourly [33] and a total determined for the energy required (MJ ha^−1^) for annual crop production from pre-seeding operations to harvest. For feeding (included processing, delivery and hauling), bedding and manure removal, the actual daily or periodic events were timed, the amounts of energy expended calculated using the hourly energy utilization rate (MJ h^−1^) for the equipment combination used [33] for embodied, fuel and lubrication energy, then totaled and expressed on an animal unit basis for each specific classification of animal units (MJ hd-d^−1^).

Energy used in the manufacture and transportation of seed, fertilizer and herbicides used were determined (Table A4) for each crop and feed combination in each year. The embodied energy coefficients for seed and herbicide were from Nagy [32,33] and Zentner *et al.* [35], and fertilizer from Snyder *et al.* [36]. Energy (MJ kg^−1^) was converted to a diesel fuel equivalent (MJ L^−1^) and then to CO_2 _equivalent (kg CO_2_e kg^−1^) for the specific input [32]. For each crop or feed all energy sources (inputs, equipment and production activities) were summed on per ha basis (kg CO_2_e ha^−1^).

Sources of direct and indirect nitrous oxide emission from cropping systems and pastures are summarized in Table A5. Methods for calculation of N_2_O generally follow the outline for defining nitrogen fractions and emission factors for crops on an ecodistrict basis as described by Rochette *et al.* [37]. The base emission factor for Ecodistrict 737 incorporates the factors for tillage, topography, irrigation and soil texture typical of the Bowden to Wetaskiwin farming areas of Alberta. Irrigation was not used and was not a consideration. No manure was applied to farm lands. Manure from grazing animals is not considered as part of the crop input component [37], and was included in the animal component for CH_4_ and N_2_O emissions from manure. N mineralization was assumed be at steady-state, therefore net mineralization was equal to 0.0 and soil mineralized-N not added to the sum of crop N-inputs. Relevant information for the ecodistrict as supplied by Worth and Desjardins [38] were P/PE = 0.65, Emission factor _soil_ = 0.0095 kg N_2_O-N kg^−^^1^ N.

Fertilizer-N applied and above ground residue and root contributions were determined and summed as direct emissions. Residue and root-N were quantified using actual data for residue dry matter, root mass and N concentration or using methods and ratios supplied by Janzen *et al*. [39] for product: above ground residue: root and appropriate N concentrations. All residue-N and root-N was assumed to be returned to the soil each year for annual crops, but only 10% of perennial pasture and hay residue was included in crop N-inputs [39]. Root mass of all perennial pasture and hay stands was assumed to be 10,000 kg ha^−1^ as approximated in smaller paddock and plot sites at Lacombe [40]. Indirect N_2_O-N emission consisted of leaching of fertilizer-N and root and residue-N that had mineralized within the year. In this case a leaching fraction (FRAC) _Leach_ = 0.19 kg N ha^−1^ of N-inputs and volatilization of applied fertilizer-N of = 0.10 kg N ha^−1^ of fertilizer-N inputs. Emission factor for leaching (EF _Leach_) was 0.0075 kg N_2_O kg^−1^ N ha^−1^ and the emission factor for volatilization = 0.01 kg N_2_O kg^−1^ N ha^−1^. 

### 2.5. Scaling

Enteric CH_4_ and manure CH_4_ and N_2_O were expressed in kg head^−1^ d^−1^ and multiplied by 23 for CH_4_ and 298 for N_2_O and then by the number of days in each feeding period for each cattle class to give kg CO_2_e head^−1^ period^−1^. Carbon dioxide equivalents from enteric fermentation and manure were then summed across feeding periods for each cattle class to give kg CO_2_e head^−1^ yr^−1^ or kg CO_2_e head^−1^ period^−1^ for calf- and yearling-fed steers and heifers where period refers to the days from birth to harvest. These values were then scaled to a herd size of 160 cows assuming 85% calves weaned of cows exposed to breeding (n = 136), 10% culled cows in the fall due to reproductive failure (n = 16), 5% cows culled in the spring due to failure to deliver a live calf, calving difficulty or temperament (n = 8), 15% replacement heifers to replace the culled cows and keep the herd growth at zero (n = 24), a 25:1 cow:bull ratio (n = 6 bulls), six young replacement bulls, six cows to produce the six replacement bulls and 68 slaughter steers and 44 slaughter heifers that is based on 136 calves with 24 females being kept as herd replacements. These proportions are based on the Alberta Cow-calf Audit [41]. The actual live slaughter and carcass weight for the calf- and yearling-fed steers were taken from López-Campos *et al.* [27] and multiplied by 68 to obtain total beef sold for steers. Live slaughter was 10% lower and dressing percentage 0.5 percentile points lower in heifers than steers [23,28] and these values were used to calculate the live slaughter and carcass weight of heifers that were then multiplied by 44 to obtain total weight of beef sold from slaughter heifers. The diet ingredient composition (DM basis) and DMI within each feeding period and cattle category (Table A1 and Table A2 ) were used to calculate total feed or crop ingredients (kg DM) used. These values were then multiplied by the appropriate energy CO_2_ and cropping N_2_O emission coefficient within year (Table A6) to given total kg CO_2_e from energy use and cropping.

## 3. Results and Discussion

The GHG intensity or carbon footprint of beef ranged from 11.63 to 13.22 kg CO_2_e kg^−1^ live weight or from 19.87 to 22.52 kg CO_2_e kg^−1^ carcass weight and depended on beef production strategy (Table A7). Beauchemin *et al.* [11] simulated beef and cropping production over eight years using the HOLOS whole-farm model [42] and reported a carbon footprint of 13.04 kg CO_2_e kg^−1^ live weight and 21.73 kg CO_2_e kg^−1^ carcass weight for implanted feeder cattle harvested at 16.5 mo of age. Similar cattle from our study are estimated to have carbon footprints of 12.43 CO_2_e kg^−1^ live weight and 21.20 kg CO_2_e kg^−1^ carcass weight which are 4.7 and 2.4% lower than those presented by Beauchemin *et al.* [11]. The higher values in Beauchemin *et al*. [11] are likely due to the higher enteric emission factor (7.0% *vs.* 6.5%) used for cows, heifers and bulls on a poor quality mixed hay diet compared to the diets for the same categories of animals used in our study (Table A1; TDN 57–65%). The GHG intensity of beef at the farm gate varies considerably from 16–27 kg CO_2_e kg^−1^ carcass weight [43,44,45] in European studies to as high as 44 kg CO_2_e kg^−1^ carcass weight for Brazilian beef that included land use change due to deforestation ([9]; 28 kg CO_2_e kg^−1^ carcass weight not including land use changes), reflecting not only differences in farming systems but also differences in assumptions, approaches and equations used to calculate GHG emissions. The large emissions associated with beef production in Brazil are not surprising considering a calving interval of 20 mo and 3–4 yr to slaughter [9] compared to a calving interval of 12 mo and average age to slaughter of 18–19 mo of age for Canadian beef production systems [19,41].

**Figure 1:**
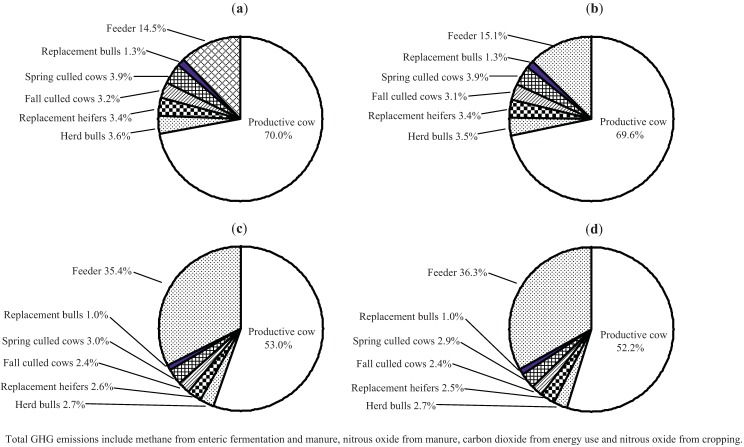
Breakdown of total greenhouse gas (GHG) emissions resulting from hormone free and growth implanted calf-fed and yearling-fed beef production systems (CO_2_) equivalents, 160 cow-herd assumed). (**a**) Calf-fed, Hormone Free Animal GHG emissions = 922,107 kg CO_2_e. (**b**) Calf-fed, growth implanted Animal GHG emissions = 928,344 kg CO_2_e. (**c**) Yearling-fed, Hormone Free Animal GHG emissios = 1,219,659 kg CO_2_e. (**d**) Yearling-fed, Growth Implanted Animal GHG emissions = 1,237,082 kg CO_2_e.

The breakdown of GHG emissions by source (Table A7, illustrated in Figure 1) showed that 53–54% of GHG emissions result from enteric CH_4_, 20–22% from manure N_2_O, 11% from cropping N_2_O, 9–9.5% from energy use and 4–6% from manure CH_4_, regardless of beef production strategy. These proportions are somewhat different than those presented by Beauchemin *et al*. [11] which were 63%, 23%, 4%, 5% and 5% for enteric CH_4_, manure N_2_O, cropping N_2_O, energy use CO_2_ and manure CH_4_, respectively. The primarily reason for these differences are because in our study (1) a enteric methane emission factor of 6.5% [22] was used for wintering cow and bull diets instead of 7.0% for poorer quality mixed hay diets, (2) more fertilizer N and equipment time was used to produce the crops for feeding cattle, and (3) the cropping mix used was more complicated than the barley grain, hay and barley silage used in the simulated southern Alberta study. However, the relative proportion of GHG emission from enteric CH_4_ was well within the range of 40–63% reported for North American beef production systems [11,46].

**Figure 2:**
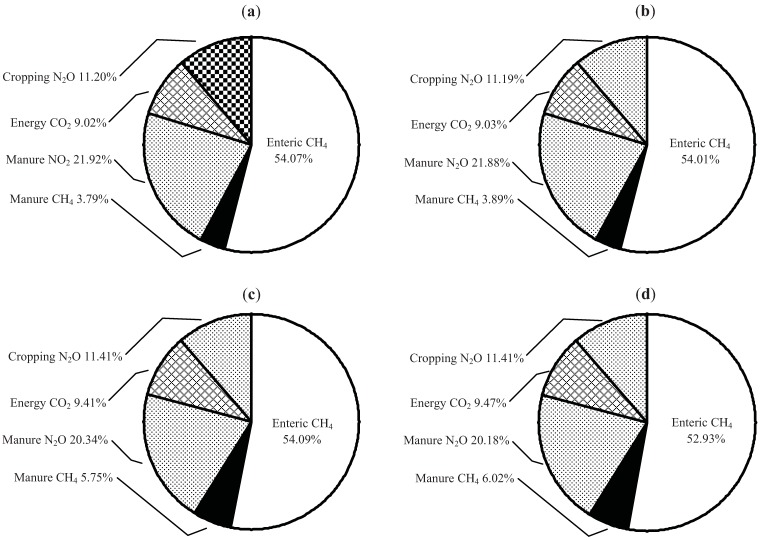
Breakdown of greenhouse gas emissions by source resulting from unimplanted and implanted calf-fed and yearling-fed beef production systems (CO_2_) equivalents; 160 cow-herd assumed). (**a**) Calf-fed, Hormone Free. (**b**) Calf-fed, Implanted. (**c**) Yearling-fed, Hormone Free (**d**) Yearling-fed, Implanted.

Total animal emissions of GHG, expressed as kg CO_2_e hd^−1^ yr^−1^, varied by year and ranged from 3,394 to 3,877 for beef cows, 4,101 to 4,912 for breeding bulls and 980 to 1,124 for replacement heifers. Non-implanted calf-fed steers ranged from 910 to 1,000 while implanted calf-fed steers ranged from 935 to 1,066 CO_2_e hd^−1^ from weaning to slaughter at the farm gate. Total animal emissions from weaning to slaughter were 3.0–3.3 times higher for yearling-fed compared with calf-fed steers and ranged from 2,754 to 3,312 for non-implanted steers and from 2,958 to 3,345 for implanted steers. Total emissions for feeder heifers were 2.9% lower than for feeder steers. The variation in GHG emissions within cattle category was due to yearly differences in diet TDN, CP and DMI, and time on each diet. The beef cow was responsible for 77% of the GHG emissions in the calf-fed and 58% in the yearling-fed beef production system (Table A7; Figure 2). Feeder steers and heifers accounted for the second highest proportion of total GHG, with the calf- and yearling-fed systems producing 15% and 35–36% of the total GHG, respectively. The proportion of GHG resulting from replacement heifers, replacement bulls or herd bulls was small (1–3.6%) and depended on beef production system. The proportions for the calf-fed system are similar to those presented by Beauchemin *et al.* [11] for feeders slaughtered at 16.5 mo of age. Total emissions to produce a youthful beef animal from conception through to harvest at the farm gate ranged from 8.2 to 11.0 t CO_2_e. To put this into perspective, the GHG emission from a mid-sized car are about 6.8 to 7.0 t CO_2_e yr^−1^ which includes the car’s direct emissions of CO_2_, along with CO_2_ emitted in producing and distributing fuel but not from manufacturing the car (U.S. Department of Energy and U.S. Environmental Protection Agency, www.fueleconomy.org).

The carbon footprint for hormone free feeder calves slaughtered at 11–14 mo of age and 518 kg was 12.23 kg CO_2_e kg^−1^ live weight, while that for implanted feeder calves slaughtered at 11–14 mo of age and 558 kg was 11.63 kg CO_2_e kg^−1^ live weight (Table A7). Similarly the carbon footprint of hormone free feeders harvested at 19–23 mo of age and 669 kg was 5.1% more GHG intensive than implanted feeders harvested at 19–23 mo of age and 725 kg (13.22 *vs.* 12.55 kg CO_2_e kg^−1^ live weight). The yearling-fed beef production systems were 6.3–7.5% more GHG intensive than the calf-fed beef production systems, and the management practices of age to harvest and use of growth promotants were additive since the carbon footprint of a hormone free yearling-fed beef was 11.8–12.0% higher than implanted calf-fed beef. Similar results have been reported by Pelletier *et al*. [7] where implanted feeders harvested at 21–23 mo of age and 636 kg were 8.6% more GHG intensive than implanted feeders harvested at 16–17 mo of age and 636 kg (16.2 *vs.* 14.8 kg CO_2_e kg^−1^ live weight). These results are consistent with previous research that has shown that higher quality feeds and increased growth rates as seen during the feedlot finishing period reduce enteric CH_4_ and manure N_2_O emissions [43,45,46]. Comparison of carbon footprints based on kg CO_2_e kg^−1^ live weight or carcass weight likely under-estimates the true difference in carbon footprint between beef production systems since efficiency is also a function of time, where improved productivity results in more beef in a set period of time per unit of animal input and waste [2,3]. When adjusted for time, the carbon footprint of the calf-fed beef production systems in our study were 73.9 to 76.1% lower than those from the yearling-fed beef production systems, and calf-fed implanted beef production was 85% lower than hormone free yearling-fed beef production.

Feed and land requirements to produce 112 youthful slaughter cattle in each of four beef production systems are presented in Table A8. Implanted calf-fed beef required 0.8% more total feed ingredients and 0.4% more total land than hormone free calf-fed beef, while implanted yearling-fed beef required 1.5% more total feed and 1.0% more land than hormone free yearling-fed beef. The yearling-fed beef production system required 22.7–23.2% more total feed ingredients and 21.0–21.5% more total land than a calf-fed beef production system. As with enteric CH_4_ and manure N_2_O, the “hot-spots” in GHG emissions due to energy use and cropping N_2_O are associated with the fresh forage and barley silage fed to the mature cow and the fresh forage, barley silage and barley grain used to background and finish feeder cattle in the yearling-fed beef production systems.

### 3.1. Land Use Efficiency

Yearling systems required approximately 85.8 ha more land to complete the production cycle than calf-fed systems. However, the proportion of land composed of annual and perennial crops was identical and carcass productivity per unit land area was similar between yearling and calf fed systems (2.0 to 2.5% mean difference). Carcass productivity per unit land area was similar because carcass size of yearling fed systems averaged 31% heavier than the calf-fed systems (Table A7). Implanted compared to non-implanted carcass output per unit area was 6.9% and 7.7% larger within calf-fed and yearling-fed systems, respectively, because implanted carcasses were about 9% heavier (Table A7). The more intensive Parkland system produced 7.8 times the carcass weight per unit area compared to a more extensive Southern Alberta system described by Beauchemin *et al*. [11], which was based on native range and dryland crop production. The difference was the 2040 ha of native range [11] required for the cow herd compared to 237 ha pasture in the current study (Table A9 and Table A10). In total the Parkland systems in this study had carcass outputs averaging 140.2 kg ha^−1^ compared to 18 kg ha^−1^ in the Southern Alberta study.

### 3.2. Soil Organic C-Sequestration

Soils under crop management (e.g., cereals and oilseeds) that has occurred for 100 years after breaking may be at a soil organic carbon (SOC) equilibrium level typical of that management, but with lower SOC stores. Conversion of cropland to grassland may result in increasing SOC stores that occur rapidly over the first 20 years, but may continue at a much slower rate for up to 50 or 100 years [47,48]. The majority of SOC accumulation may occur within the first decade of grassland conversion unless additional practices such as application of fertilizer-N occur in concert with the conversion [49]. Soil organic matter gains on conversion of cropland to grassland will be composed of relatively high proportions of light fraction SOC, which may be highly degradable and subject to loss under some conditions [50,51,52]. The reverse process of SOC loss after grassland conversion to cropland follows a trajectory of high annual SOC loss-rates immediately after conversion, then decreasing rates with time [47,48]. Land used in the current study has been under cultivation for over 100 years, but has been in rotation between forage, cereal and oilseed crops frequently over that period. Thus, the trends in SOC may be increasing for the forage and pasture stands and at equilibrium or decreasing for lands used for cereal grain and silage [53,54]. The amounts of land and short period of the study prevented a detailed assessment of changes in soil carbon and effectiveness as a greenhouse gas sink. Therefore gain and loss of SOC was attributed to each land type based on literature, available measurement, management and time from conversion from or to cropland and grassland (*i.e.*, stand age).

Using the Century model, the Canadian National Inventory Report [48] estimates for conversion of cropland to grassland in the Prairie Parkland a linear sequestration rate of 0.55 Mg ha^−1^ yr^−1^ SOC over the first 20 yr of conversion and a mean rate of 0.2 Mg ha^−1^ yr^−1^ SOC over 100 years; a land management change from grassland to cropland or in reverse requires SOC loss of the same magnitude. Net Ecosystem CO_2_ flux (NEE) was determined annually and continuously with the Bowen Ratio Energy Balance (BREB) method on cropland converted to fertilized, grassland [55] from 2002 until 2008, including establishment, for areas used in feed production of this study. Including the year of establishment a BREB annual NEE rate averaged 0.6 Mg C (2.1 Mg CO_2_) ha^−1^ yr^−1^ on this converted perennial stand. A rotation of cereals and oilseeds (2005 to 2009) in an adjacent field, broken from timothy, averaged a NEE loss of 0.45 Mg C (1.65 Mg CO_2_) ha^−1^ yr^−1^. These local sequestration and emission rates are in general agreement with the current literature [47,48,56,57] for conversion into or out of grassland. Lower sequestration rates were attributed to hay and haylage stands than new pasture (Table A9), because pasture had higher residue-C inputs. Hay, silage and grain lands had residues removed from fields for feeding and therefore had lower direct C inputs to soil than pasture [58]. However, no C grown within the farm or study boundaries left the farm. No C was transported off the farm in the form of feed, fiber or manure.

System net emissions per unit area were reduced by 11% and 16% for calf-fed and yearling fed systems, respectively, when SOC loss and sequestration were included in the determination of greenhouse gas intensity (Table A9 and Table A10). The difference between the calf- and yearling-fed systems was the use of recently converted, highly productive pasture in the yearling-fed system (Table A9), to which was attributed the highest SOC-sequestration rate as well as more silage and grain to which slightly lower loss-rates occurred. The native pasture used in the study of Beauchemin *et al*. [11] may have been at equilibrium as it had not been cultivated, although the carbon stores may have been very high. Soil carbon stores [59] to a 30-cm depth under long-term stands of unfertilized tame grass-legume, native grass and old grass mixed species were 107.4, 90 and 76.2 Mg C ha^−1^ for a Dark Brown soil at Scott, SK. Comparable side by side cropped sequences to each grassland treatment at Scott, SK were 65.0, 51.4 and 52.6 Mg C ha^−1^, respectively. In addition the GHG intensities, expressed as kg CO_2_e per kg carcass weight or kg CO_2_e per kg carcass weight per yr were reduced by 10.9%, 10.9%, 16.1% and 15.6% for the calf-fed non implanted, calf-fed implanted, yearling-fed non implanted and yearling-fed implanted beef production strategies, respectively. Again the difference in GHG emission reduction was due to more tame pasture used (62 ha) and thus more C-sequestration in the yearling-fed production system compared to the smaller loss-rates incurred by the extra silage (13.5 ha) and grain (7 to 10 ha).

## 4. Conclusions

This study provided an evaluation of the whole-farm greenhouse gas emissions from different beef cattle production systems using life cycle assessment and actual daily and/or monthly farm inputs and outputs. The whole-farm GHG emissions for Alberta beef production systems were at the lower range of global carbon footprints and comparable to carbon footprints for beef in North America. The inclusions of carbon sequestration from pasture and hay land use and soil organic carbon loss from annual cropping decreased the carbon footprint of beef by 11–16%. Yearling-fed compared with calf-fed production systems produce more carcass weight per animal, but use more time, feed and land and could have higher carbon footprints particularly when expressed per unit of time. Strategies to reduce GHG emissions should emphasize improving feed efficiency of the cow herd and decreasing the length of time feeder cattle are on low quality feeds. In addition land use practices that obtain more than one feed ingredient per crop (e.g., grain and straw) and optimize carbon sequestration and soil carbon change will have a significant impact on reducing GHG emissions from energy use CO_2_ and cropping N_2_O.

## Appendix

**Table A1:** Description of diets ingredient and nutrient composition, animal growth and feed intake by cattle category averaged over two years.

Cattle category	Animal used	Dayson feed	Diet Ingredient Composition (DM basis)	TDN, %	CP, %	Mid-Wt, kg	ADG, kg d^−1^	DMI, kg d^−1^
**Cow-calf pairs and cows**								
Cow-calf pairs, summer	93–99	111.0	100% MBA pasture	63.33	16.13	656.8	0.22	17.37
Cow-calf pairs pre-weaning	93–99	14.0	49.40% MBA pasture: 50.60% BS	62.98	12.76	671.9	0.21	21.73
Cows, post weaning	90–110	21.0	100% BS	60.75	13.02	674.0	1.15	10.27
Cows, winter drylot	21–42	125.5	48.17%BS:3.94%BG:2.62%OG:19.59%H:25.70%St	57.05	10.78	719.6	0.52	12.65
Cows, pre- & post-calving	95–114	93.5	78.87%BS:15.42%BG:0.69%OG:5.03%H	62.93	12.84	714.7	0.82	11.44
Total days on feed		365.0						
**Herd bulls**								
Summer grazing on-pasture	4–5	108.5	100% MBA pasture	63.33	16.13	866.0	1.03	21.48
Fall on-pasture	12–15	43.5	100%BS	60.63	13.06	921.0	0.01	10.80
Wintering on-pasture	12-15	203.0	76.64%BS:20.58%BG:2.78%H	63.32	13.22	921.0	0.36	15.25
Spring pre-grazing on-pasture	12–15	10.0	100%BS	65.66	13.55	866.0	0.01	11.07
Total days on feed		365.0						
**Replacement heifers**								
On-pasture with dam	93–99	137.0	100% MBA pasture	63.33	16.13	168.4	1.17	NA
Post-weaning, feedlot pen	77–99	24.5	84.21%BS:15.79%GS	59.97	13.01	251.3	0.60	4.35
Wintering, feedlot pen	61–72	105.5	79.14%BS:20.86%BG	65.10	12.70	291.6	0.60	6.18
Wintering, Performance Test	61–72	98.0	77.76%BS:22.24%BG	63.99	12.45	355.2	0.66	7.80
Total days on feed		365.0						
**Replacement bulls**								
On-pasture with dam	67–69	126.0	100% MBA pasture	63.31	16.12	179.3	1.07	NA
Weaning to Bull Test	67–69	32.0	100%BS	62.85	12.84	273.1	0.59	6.79
Bull Test period	67–69	117.0	33.00%BS:60.00%BG:7.00%Ps	78.20	13.64	369.8	1.49	9.24
Wintering, Performance Test	67–69	90.0	100%BS	62.85	12.84	483.9	0.59	12.04
Total days on feed		365.0						

Abbreviations are: MBA = meadow brome alfalfa; BS = barley silage; GS = MBA grass silage; BG = barley grain; OG = oat grain; H = MBA hay; St = barley straw; Ps = protein supplement. NA, Not applicable as calf feed intake during summer grazing is included in the cow-calf pair category.

**Table A2:** Description of diets ingredient and nutrient composition, animal growth and feed intake by feeder category averaged over two years.

Feeder Category	Animal used	Days on feed	Diet Ingredient Composition (DM basis)	TDN, %	CP, %	Mid-Wt, kg	ADG, kg d^−1^	DMI, kg d^−1^
**Calf-fed, not implanted**								
Weaning to step-up	56	13.5	83.34%BS:16.66%GS	59.92	13.01	263.1	0.79	4.14
Step-up	56	34.5	18.19%BS:49.85%BG:24.90%GS:7.05%Ps	69.20	13.54	286.4	1.13	6.97
Feedlot finishing, Progeny test	56	83.0	10.38%BS:70.88%BG:9.34%GS:9.30%Ps	74.22	12.84	369.0	1.50	8.02
Feedlot finishing	56	76.0	18.87%BS:71.62%BG:9.51%Ps	75.06	14.78	484.1	1.45	8.55
**Calf-fed, implanted**								
Weaning to step-up	56	13.5	83.34%BS:16.66%GS	59.92	13.01	262.9	0.61	4.14
Step-up	56	34.5	18.19%BS:49.85%BG:24.90%GS:7.05%Ps	69.20	13.54	288.8	1.31	7.07
Feedlot finishing, Progeny test	56	83.0	10.38%BS:70.88%BG:9.34%GS:9.30%Ps	74.22	12.84	385.4	1.75	8.52
Feedlot finishing	56	76.0	18.87%BS:71.62%BG:9.51%Ps	75.06	14.78	519.1	1.67	9.04
**Yearling-fed, not implanted**								
Weaning to fall pasture	56	13.5	83.34%BS:16.66%GS	59.92	13.01	261.7	0.71	4.52
Fall pasture	56	41.5	100% MBA	55.82	7.95	267.9	0.18	3.65
Winter backgrounding	56	191.0	66.27%BS:20.55%GS:5.18%BG:2.73%OG:5.27%St	61.76	12.28	358.8	0.92	7.80
Summer pasture	56	66.0	100% MBA	59.39	12.31	463.8	0.50	14.83
Step-up	56	22.0	33.96%BS:60.24%BG:5.80%Protein sup.	71.32	13.42	502.1	1.90	9.83
Feedlot finishing, Progeny test	56	76.5	16.37%BS:77.81%BG:5.82%Protein sup.	74.57	12.66	588.3	1.71	12.50
Feedlot finishing	56	47.5	19.54%BS:74.72%BG:5.74%Protein sup.	73.40	12.86	674.0	0.90	11.74
**Yearling-fed, implanted**								
Weaning to fall pasture	56	13.5	83.34%BS:16.66%GS	59.92	13.01	264.3	1.02	4.52
Fall pasture	56	41.5	100% MBA	55.82	7.95	270.3	0.13	3.65
Winter backgrounding	56	191.0	66.27%BS:20.55%GS:5.18%BG:2.73%OG:5.27%St	61.76	12.28	366.0	0.99	8.25
Summer pasture	56	66.0	100% MBA	59.39	12.31	479.7	0.55	13.64
Step-up	56	22.0	33.96%BS:60.24%BG:5.80%Protein sup.	71.32	13.42	524.4	2.33	10.60
Feedlot finishing, Progeny test	56	76.5	16.37%BS:77.81%BG:5.82%Protein sup.	74.57	12.66	626.1	2.00	14.10
Feedlot finishing	56	47.5	19.54%BS:74.72%BG:5.74%Protein sup.	73.40	12.86	727.7	1.11	12.48

Abbreviations are: MBA = meadow brome alfalfa; BS = barley silage; GS = MBA grass silage; BG = barley grain; OG = oat grain; H = MBA hay; St = barley straw; Ps = protein supplement.

**Table A3:** Monthly mean temperature and total monthly precipitation at Lacombe, AB from 2008 to 2010.

	Mean monthly daily mean temperature, °C				Monthly precipitation, mm			
Month	2008	2009	2010	100 years average	2008	2009	2010	100 years average
January	−11.2	−10.7	−9.2	−13.5	14.6	14.3	6.7	18.2
February	−8.4	−9.7	−6.8	−10.3	26.3	9.6	0.7	16.2
March	−1.4	−7.2	1.1	−4.8	6.3	17.1	1.3	18.1
April	1.4	3.1	5.0	3.7	25.9	15.2	36.4	26.0
May	10.3	8.5	7.8	9.8	58.8	14.7	91.2	51.0
June	13.5	12.7	13.3	13.6	102.4	41.4	116.6	83.4
July	15.5	15.9	15.3	16.1	63.1	92.3	212.0	78.8
August	15.6	14.5	14.4	14.9	66.5	74.0	39.8	65.5
September	10.3	13.7	8.3	10.1	9.6	9.5	54.2	42.0
October	5.3	3.0	6.3	4.4	10.6	11.1	0.0	19.9
November	0.5	−0.1	−6.4	−4.4	0.0	0.6	20.4	15.7
December	−14.1	−16.4	−12.4	−10.6	11.4	0.0	3.6	15.5
Total					395.5	299.8	582.9	450.3

**Table A4:** Sources of carbon dioxide emissions related directly and indirectly to on-farm fossil fuel use for each crop and pasture.

Gas/Source	Fraction, emission factor, equation	Reference
**CO_2_ machine operations (kg CO_2_ ha^−1^)**	= ∑_Embodied_ +∑_Fuel_+ ∑ _Lubricants_	[32,33]
CO_2_ Embodied (kg CO_2_ ha^−1^)	=( (MJ hr^−1^_equipment unit_) / (43.99 (MJ L^−1^)) × (2.639 kg CO_2_ L^−1^) / work rate (ha h^−1^)	[32,33]
CO_2_ Fuel (kg CO_2_ ha^−1^)	=( MJ h^−1^_power unit_) / (43.99 (MJ L^−1^_diesel_)) × (2.639 kg CO_2_ L^−1^) / work rate (ha h^−1^)	
CO_2_ Lube (kg CO_2_ ha^−1^)	= (MJ h^−1^_Lube_) / (43.99 (MJ L^−1^)) × (2.639 kg CO_2_ L^−1^) / work rate (ha h^−1^)	[32,33]
Work rate (ha h^−1^) = power unit, implement and width. Fuel use rate (L h^−1^) = Diesel fuel by power unit, implement and width Lube use rate = oil and grease *etc.* (MJ h^−1^) implement and power unit combination) 43.99 MJ L^−1^ diesel fuel 2.639 kg CO_2_ L^−1^ diesel fuel		[34] [32,33]
**CO_2_ Cropping inputs (kg CO_2_ ha^−1^)**	=∑_seed_ +∑_fertilizer_ + ∑ _herbicide_	
CO_2 _Seed (kg CO_2_ ha^−1^)	= MJ kg^−1^_seed production_ / 43.99 MJ L^−1^ × 2.639 kg CO_2_ L^−1^ × seeding rate (kg ha^−1^)	[32,33]
CO_2_ Fertilizer (kg CO_2_ ha^−1^)	= MJ kg^−1^_nutrient_ / 43.99 MJ L^−1^ × 2.639 kg CO_2_ L^−1^ × nutrient rate (kg ha^−1^)	[32,33]
CO_2_ Herbicide (kg CO_2_ ha^−^^1^)	= MJ kg^−1^_herbicide_ / 43.99 MJ L^−1^ × 2.639 kg CO_2_ L^−1^ × herbicide rate (kg ha^−1^)	[32,35]
Seed production MJ kg^−1^ energy to produce 1 kg seed for purposes of crop seeding Nutrient = 53 MJ kg^−1^ urea-N; 14 MJ kg^−1^ P_2_O_5_; 9 MJ kg^−1^ K_2_O manufacture and transportation Herbicide = 373 MJ kg^−1^ ai Glyphosate and 104.5 MJ kg^−1^ ai Dicamba and 2,4-D amine and Mecoprop		[33] [36] [35]

**Table A5:** Sources of cropping related direct and indirect nitrous oxide emissions for each crop and pasture.

Gas/Source	Fraction, emission factor, equation	Reference
Nitrous oxide Sources		
N_2_O direct cropping (kg CO_2_ ha^−1^)	= ((∑_Fert-N_ + ∑ _(Residue + Root-N)_ ( kg N ha^−1^)) x EF_eco_ (kg N_2_O-N kg^−1^ N) x (44/28)	[37]
N_2_O indirect cropping (kg CO_2_ ha^−1^)	= ∑_Leach_ + ∑_Volatilization_	[22]
N_2_O indirect leaching (kg CO_2_ ha^−1^)	= (∑_Fert-N_ + ∑ _(Residue + Root-N)_ ( kg N ha^−1^)) x Frac _Leach x_ EF _Leach _(kg N_2_O-N kg^−1^ N) x (44/28))	
N_2_O indirect volatilization (kg CO_2_ ha^−1^)	= (∑_Fert-N_ ( kg N ha^−1^)) x Frac _vol_. x EF _vol_. (kg N_2_O-N kg^−1^ N) x (44/28)	[22]
∑ _(Residue + Root-N)_ Represents all residue and root-N determined and measured for annuals and 10% of residue and root-N for perennials, annually.		[39]
EF _eco_ for Ecodistrict 737 = 0.0095 (kg N_2_O-N kg N)		[38]
Frac _Leach_ for Ecodistrict 737 = 0.19 (kg N ha^−1^)		[38]
EF _Leach_ = 0.0075 (kg N_2_O-N kg N)		[22]
Frac _vol_._ = _0.10 (kg Fert. N ha^−1^)		[22]
EF _vol_. = 0.01 (kg N_2_O-N kg N)		[22]

**Table A6:** Greenhouse gas emission coefficients for energy use ^x^ and crop nitrous oxide ^y^ (N_2_O) by crop type and year.

Crop Type	2008–09		2009–10	
	Energy CO_2_	Crop N_2_O	Energy CO_2_	Crop N_2_O
Summer pasture, cow-calf pair, kg CO_2_e hd.d^−1^	0.11643	1.26118	1.78865	3.27245
Summer pasture, herd bull, kg CO_2_e hd.d^−1^	0.11168	1.20976	1.74362	3.19006
Fall pasture + straw, weaned calf, kg CO_2_e hd.d^−1^	0.10576	0.18609	0.09688	0.18268
Summer pasture, yearling, kg CO_2_e hd.d^−1^	1.71257	2.18789	1.91910	2.87737
Barley silage, kg CO_2_e kg^−1^ DM	0.08731	0.08258	0.08080	0.07322
Barley grain, kg CO_2_e kg^−1^ DM	0.07737	0.07066	0.06933	0.06044
Oat grain, kg CO_2_e kg^−1^ DM	0.12109	0.10597	NA	NA
Meadow brome alfalfa hay, kg CO_2_e kg^−1^ DM	0.10581	0.07763	NA	NA
Meadow brome alfalfa silage, kg CO_2_e kg^−1^ DM	0.09858	0.07763	NA	NA
Barley straw, kg CO_2_e kg^−1^ DM	0.09570	0.07362	0.08809	0.06038
Protein supplement plus minerals^z^, kg CO_2_e kg^−1^ DM	0.06600	0.05400	0.06600	0.05400
Barley straw for bedding, kg CO_2_e kg^−1^ DM	0.05604	0.07362	0.05131	0.06038

^x^ Energy use includes embodied energy in equipment for field operations, baling, hauling, feed processing, feeding, bedding and manure removal; crop inputs such as fertilizer, herbicide and seed; fuel and lubrication for field operations, baling, hauling, feed processing, feeding, bedding and manure removal. ^y^ Crop N_2_O includes emissions of N_2_O from soil, fertilizer, roots and residue. NA Not fed in 2009–10 so no coefficients were determined. ^z^ Greenhouse gas mission intensities for protein supplement are based on Dyer *et al.* [60] and Quantification Protocol for Emissions Reduction from Dairy Cattle http://environment.gov.ab.ca/info/library/8255.pdf where the average intensity for canola is 0.8 kg CO_2_e per kg DM. The authors assumed a 85:15 split between canola used for oil and canola meal used for livestock feeding, and a 55:45 split between GHG from energy use and GHG from cropping N_2_O.

**Table A7:** Greenhouse gas emission by cattle category and source, and overall GHG intensity for non-implanted (NI) and implanted (IMP) calf- and yearling-fed beef production systems (assuming 160 cows) ^y^.

Cattle category	Productive cows	Herd Bulls^z^	Repl. heifers	Fall cull cows	Spring cull cows	Repl. bulls	Calf-fed NI	Calf-fed IMP	Year-fed NI	Year-fed IMP
Number of cattle	142	6	24	16	8	6	112	112	112	112
Period, d	365	365	365	112	365	365	207	207	454	454
Live slaughter weight, kg hd^−1^	NA	866	NA	651	651	NA	518.3	557.5	668.8	725.3
Carcass weight, kg hd^−1^	NA	493.6	NA	371	371	NA	302.0	328.9	395.2	432.4
Total live weight sold, kg	0	1,730	0	10,416	5,208	0	58,045	62,435	74,911	81,233
Total carcass weight sold, kg	0	986	0	5,936	2,968	0	33,827	36,840	44,258	48,427
**Greenhouse gas emissions from enteric fermentation and manure handling, storage and land application from beef herd**										
Enteric CH_4_, kg CO_2_e hd^−1^ period^−1^	2,530.24	2,976.78	754.52	955.70	2,530.24	1,180.61	541.84	566.97	1,871.31	1,936.36
Manure CH_4_, kg CO_2_e hd^−1^ period^−1^	58.91	66.08	16.33	21.10	58.91	21.93	221.81	232.64	535.57	574.71
Manure N_2_O, kg CO_2_e hd^−1^ period^−1^	1,038.68	1,465.65	280.40	485.60	1,038.68	467.86	180.60	189.27	591.40	604.34
Sub-total, kg CO_2_e hd^−1^ period^−1^	3,627.83	4,508.51	1,051.25	1,462.45	3,627.83	1,670.40	944.24	988.87	2,998.28	3,115.41
Enteric CH_4_, kg CO_2_e period^−1^	359,294	17,861	18,109	15,291	20,242	7,084	60,686	63,500	209,586	216,873
Manure CH_4_, kg CO_2_e period^−1^	8,365	397	392	338	471	132	24,843	26,055	59,984	64,367
Manure N_2_O, kg CO_2_e period^−1^	147,493	8,794	6,730	7,770	8,309	2,807	20,227	21,198	66,237	67,686
Sub-total, kg CO_2_e period^−1^	515,151	27,051	25,230	23,398	29,023	10,022	105,755	110,754	335,807	348,926
**Greenhouse gas emissions from energy use and cropping**										
Energy use CO_2_, kg CO_2_e period^−1^	56,530	2,585	3,144	1,707	3,185	1,182	14,845	15,504	46,483	48,764
Cropping N_2_O, kg CO_2_e period^−1^	74,169	3,300	2,939	4,062	4,179	1,093	13,555	14,134	49,417	51,440
Sub-total, kg CO_2_e period^−1^	130,700	5,885	6,083	5,769	7,363	2,276	28,400	29,639	95,900	100,204
Total live weight sold, kg							75,399	79,789	92,265	98,587
Total carcass weight sold, kg							43,717	46,731	54,148	58,317
Total enteric CH_4_, kg CO_2_e							498,566	501,380	647,466	654,752
Total manure CH_4_, kg CO_2_e							34,936	36,149	70,078	74,461
Total manure N_2_O, kg CO_2_e							202,129	203,101	248,139	249,588
Total energy CO_2_, kg CO_2_e							83,178	83,838	114,816	117,098
Total crop N_2_O, kg CO_2_e							103,297	103,877	139,160	141,182
Total GHG emissions, kg CO_2_e							922,107	928,344	1,219,659	1,237,082
GHG Intensity, kg CO_2_e kg^−1^ live weight							12.23	11.63	13.22	12.55
GHG Intensity, kg CO_2_e kg^−1^ carcass weight							21.09	19.87	22.52	21.21
GHG Intensity^y^, kg CO_2_e kg^−1^ live weight yr^−1 ^							13.10	12.46	23.07	21.90
GHG Intensity^y^, kg CO_2_e kg^−1^ carcass weight yr^−1 ^							22.60	21.28	39.31	37.02

^y^ Calf- and yearling-fed feeders consisted of 66 steers and 44 heifers, and their means and totals are based on weighted averages. ^z^ One-third of live slaughter and carcass weight is assigned to beef production each year since all breeding bulls are culled every three years.

**Table A8:** Feed and land use for non-implanted (NI) and implanted (IMP) calf- and yearling-fed beef production systems (assuming 160 cows).

Cattle category	Productive cows	Herd bulls^y^	Repl. heifers	Fall cull cows	Spring cull cows	Repl. bulls	Calf-fed NI	Calf-fed IMP	Year-fed NI	Year-fed IMP
Number of cattle	142	6	24	16	8	6	112	112	112	112
Period, d	365	365	365	112	365	365	207	207	454	454
**Feed resources used for beef herd averaged over two years**										
Fresh forage ^x^, head.d	16,744	651	0	1,792	943	0	0	0	12,040	12,040
Fresh forage, kg DM period^−1^	295,774	13,968	0	30,868	16,663	0	0	0	123,740	116,249
Barley silage, kg DM period^−1^	279,762	17,713	28,827	0	15,761	9,946	32,237	33,650	148,788	159,470
Barley grain, kg DM period^−1^	33,714	3,836	7,306	0	1,899	3,892	117,280	123,556	152,629	167,570
Oat grain, kg DM period^−1^	8,049	0	0	0	453	0	0	0	4,905	5,083
MBA hay, kg DM period^−1^	59,974	518	0	0	3,379	0	0	0	36,987	38,329
Grass silage, kg DM period^−1^	0	0	418	0	0	0	13,207	13,605	1,263	1,263
Barley straw, kg DM period^−1^	48,166	0	0	0	2,714	0	0	0	7,921	8,587
Protein suppl., kg DM period^−1^	0	0	0	0	0	454	15,694	16,534	11,126	12,244
Straw for bedding, kg DM period^−1^	52,343	2,607	2,517	0	2,949	578	23,355	23,355	52,014	52,014
**Feed resources used to produce calf-fed and yearling-fed steers from birth to slaughter averaged over two years**										
Total fresh forage ^x^, head.d							20,130	20,130	32,170	32,170
Total fresh forage, kg DM							357,274	357,274	481,014	473,523
Total barley silage ^y^, kg DM							384,247	385,660	500,797	511,480
Total barley grain ^y^, kg DM							167,928	174,204	203,277	218,218
Total oat grain ^y^, kg DM							8,503	8,503	13,408	13,585
Total MBA hay ^y^, kg DM							63,871	63,871	100,858	102,200
Total MBA silage ^y^, kg DM							13,625	14,022	1,680	1,680
Total barley straw ^y^, kg DM							50,879	50,879	58,800	59,466
Total protein suppl. ^y^, kg DM							16,148	16,988	11,580	12,698
Total straw for bedding ^y^, kg DM							84,349	84,349	113,007	113,007
Total all feed, t DM							1,147	1,156	1,484	1,506
Land requirements, ha							318.5	319.7	403.4	407.3
Land productivity, ha t^−1^ live weight							236.7	249.5	228.7	242.1
Land productivity ^z^, ha t^−1^ live weight yr^−1^							253.6	267.3	399.1	422.4

^x^ In 2008-09, carrying capacity were 68.81, 560.00 and 114.71 hd.d ha^−1^ for cow-calf pairs plus bulls grazing summer pasture, calves grazing fall pasture and yearling grazing summer pasture, respectively. In 2009–10, carrying capacity were 100.78, 550.00 and 160.22 hd.d ha^−1^ for cow-calf pairs plus bulls grazing summer pasture, calves grazing fall pasture and yearling grazing summer pasture, respectively. ^y^ In 2008, crop yields for barley silage, barley grain, barley straw for feed and bedding, meadow-brome alfalfa hay, grass silage and oats were 9,700, 6,100, 4,795, 8,000, 8,000 and 3,850 kg DM ha^−1^, respectively. In 2009, crop yields for barley silage, barley grain, and barley straw for feed and bedding were 9,000, 5,850, 4,786 kg DM ha^−1^, respectively. ^z^ Days to harvest averaged 391 and 637 d for the calf-fed yearling-fed beef production systems, respectively.

**Table A9:** Land areas by crop type with annual C-sequestration and CO_2_ sequestration and emission coefficients.

Crop Complex	Age ^Z^ yr	Calf-Fed		Yearling-Fed		Gain or loss ha^−1^	
		Non implanted	Implanted	Non implanted	Implanted	Sequestration rate, Mg ha^−1^ yr^−1^	
		Land area utilized by systems, ha					
						C	CO_2_
Hay & Haylage	5 < 10	9.68	9.68	12.82	12.99	0.2	0.730
Old pasture	110–20	237.40	237.40	237.40	237.40	0.2	0.730
New pasture	<10	0.00	0.00	62.14	62.14	0.5	1.830
Silage (loss)	5–15	41.20	41.25	53.56	54.70	0.3	−1.098
Grain(loss)	5−15	30.32	30.37	37.47	40.05	0.3	−1.098
Total		318.60	318.70	403.39	407.28		

^Z^ Average time from establishment of perennial stands on cropland or conversion of grassland to cropland.

**Table A10:** Annual sequestration or emission of CO_2_ by soil for respective land areas used by each system and impacts on system greenhouse emission intensity.

Crop Complex	Calf-Fed		Yearling-Fed	
	Non implanted	Implanted	Non implanted	Implanted
	kg CO_2_ yr^−1^			
Hay and Haylage	7,086	7,086	9,384	9,509
Old pasture	173,777	173,777	173,777	173,777
New pasture	0.0	0.0	113,716	113,716
Silage (loss)	−45,238	−45,293	−58,809	−60,061
Grain(loss)	−33,291	−34,444	−41,142	−43,975
Total system net sequestration	102,334	101,126	196,926	192,966
System Net Emission (kg CO_2_ yr^−1^)	819,773	827,218	1,022,733	1,044,116
System Net Emission/ ha (kg CO_2_ yr^−1^ ha^−1^)	2,573	2,596	2,535	2,564
Live wt. (kg )	75,399	79,789	92,265	98,587
Carcass wt. (kg)	43,717	46,731	54,148	58,317
Live wt. (kg ha^−1^)	236.7	250.4	228.7	242.1
Carcass wt. (kg ha^−1^)	137.2	146.6	134.2	143.1
GHG Intensity, kg CO_2_ kg^−1^ live weight	10.9	10.4	11.1	10.6
GHG Intensity, kg CO_2_ kg^−1^ carcass weight	18.8	17.7	18.9	17.9
GHG Intensity, kg CO_2_ kg^−1^ live weight yr^−1^	11.7	11.1	19.4	18.5
GHG Intensity, kg CO_2_ kg^−1^ carcass weight yr^−1^	20.2	19.0	33.0	31.2
